# Therapeutic Potential of Phosphodiesterase Inhibitors against Neurodegeneration: The Perspective of the Medicinal Chemist

**DOI:** 10.1021/acschemneuro.0c00244

**Published:** 2020-05-13

**Authors:** Giovanni Ribaudo, Alberto Ongaro, Giuseppe Zagotto, Maurizio Memo, Alessandra Gianoncelli

**Affiliations:** † Department of Molecular and Translational Medicine, University of Brescia, Viale Europa 11, 25123 Brescia, Italy; ‡ Department of Pharmaceutical and Pharmacological Sciences, University of Padova, Via Marzolo 5, 35131 Padova, Italy

**Keywords:** PDE, sildenafil, tadalafil, neurodegeneration, Alzheimer’s disease, multi-target-directed ligands

## Abstract

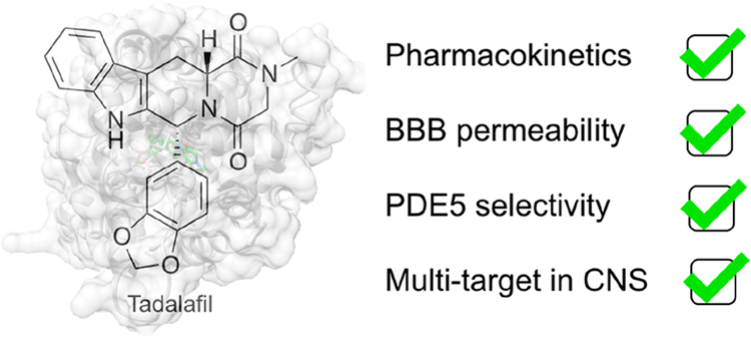

Increasing human life expectancy prompts the development of novel remedies for cognitive decline: 44 million people worldwide are affected by dementia, and this number is predicted to triple by 2050. Acetylcholinesterase and *N*-methyl-d-aspartate receptors represent the targets of currently available drugs for Alzheimer’s disease, which are characterized by limited efficacy. Thus, the search for therapeutic agents with alternative or combined mechanisms of action is wide open. Since variations in 3′,5′-cyclic adenosine monophosphate, 3′,5′-cyclic guanosine monophosphate, and/or nitric oxide levels interfere with downstream pathways involved in memory processes, evidence supporting the potential of phosphodiesterase (PDE) inhibitors in contrasting neurodegeneration should be critically considered. For the preparation of this Review, more than 140 scientific papers were retrieved by searching PubMed and Scopus databases. A systematic approach was adopted when overviewing the different PDE isoforms, taking into account details on brain localization, downstream molecular mechanisms, and inhibitors currently under study, according to available *in vitro* and *in vivo* data. In the context of drug repurposing, a section focusing on PDE5 was introduced. Original computational studies were performed to rationalize the emerging evidence that suggests the role of PDE5 inhibitors as multi-target agents against neurodegeneration. Moreover, since such compounds must cross the blood–brain barrier and reach inhibitory concentrations in the central nervous system to exert their therapeutic activity, physicochemical parameters were analyzed and discussed. Taken together, literature and computational data suggest that some PDE5 inhibitors, such as tadalafil, represent promising candidates.

## 1 Introduction

In the context of cognitive decline, the search for novel efficient diagnostic tools and treatments is wide open, since the human population is constantly aging due to increasing life expectancy. In parallel, continuous improvements in understanding the dynamic processes that underlie memory consolidation and, on the other side, pathological cognitive changes in the brain are being achieved.^1^ According to recent estimates, 44 million people currently live with dementia worldwide, and this number is predicted to triple by 2050. At that stage, the annual cost of Alzheimer’s disease (AD) in the USA would exceed US$600 billion.^2^ AD symptoms affect memory, attention, personality, intellect, and speech. Its hallmarks consist in amyloid plaques in the brain and a progressive degeneration of cholinergic innervation of the hippocampus and cerebral cortex.^3^ Concerning therapeutic options, two classes of small molecules are currently available.^4^ Acetylcholinesterase (AChE) inhibitors (donepezil, rivastigmine, galantamine) act by enhancing acetylcholine levels, but unsatisfying efficacy and adverse side effects limit their use.^5^ Memantine belongs instead to the second class of compounds, and it targets *N*-methyl-d-aspartate (NMDA) receptors, with modest effects on cognition in moderate/severe AD.^3^

A strategy for developing novel agents to restore memory function is represented by the inhibition of phosphodiesterase (PDE) activity with small molecules. PDEs are a family of enzymes encompassing 11 classes, which will be covered in the following section of this Review, that physiologically act by hydrolyzing cyclic nucleotide-based second messengers to their corresponding linear derivatives.^3^ By elevating 3′,5′-cyclic adenosine monophosphate (cAMP) and 3′,5′-cyclic guanosine monophosphate (cGMP) and/or influencing nitric oxide (NO) levels, PDE inhibitors interfere with several pathways that have been reported to be relevant in learning functions in animal models of impaired cognition.^6,7^

In this context, growing evidence suggests that some inhibitors, especially those interfering with the PDE5 isoform, may play a role in influencing cognition-related neural activity and be effective against central nervous system (CNS)-related diseases.^8−10^ For the preparation of this paper, more than 140 research papers and reviews were considered. Scientific contributions were retrieved by searching PubMed (www.ncbi.nlm.nih.gov/pubmed/) and Scopus (www.scopus.com) databases using keywords such as “phosphodiesterase”, “PDE5”, “sildenafil”, “tadalafil”, natural compounds”, “neurodegeneration”, “Alzheimer’s disease”, and their combinations. Research papers and reviews published in the 2000–2020 time frame were considered and screened. Particular attention was dedicated to the contributions reporting preclinical and clinical data on PDE inhibitors.

In the first part of this Review, an overview of the current evidence on the therapeutic potential of isoform-selective PDE1–11 inhibitors against neurodegeneration will be presented, providing updated insights about the small molecules currently under development and the underlying molecular mechanisms. As anticipated, particular attention will be dedicated to PDE5 inhibitors, which have been used for decades in clinical practice with other indications and are known for their overall safety and good pharmacokinetic properties. In the second part of the Review, a closer look at this topic from the point of view of the medicinal chemist will be provided. Chemical aspects of selected natural and synthetic PDE5 inhibitors will be discussed in more detail: computational tools, such as molecular docking and pharmacokinetic properties prediction, will support the exploration of their potential as multi-target CNS drugs.

## 2 Targeting PDE Isoforms in the CNS

Signaling pathways involving cAMP and cGMP are regulated by several enzymes. Transmembrane and soluble adenylyl cyclases (ACs) are responsible for the synthesis of cAMP in brain, while cGMP is synthesized by particulate guanylyl cyclase (pGC).^11,12^ On the other hand, cAMP and cGMP are degraded by PDEs. The interest in PDEs as targets to ameliorate age-related brain conditions first relies on the hypothesis that a breakdown in cyclic nucleotide synthesis/degradation may contribute to the onset of the disease.^11^ Moreover, altered cyclic nucleotide signaling has been previously connected with aging, and this may be caused by reduced AC activity and/or by a variation in PDEs activity or expression, as observed in animal models, and brain region-specific alterations in cyclic nucleotide signaling are thought to contribute to the onset and progression of AD.^13^ In particular, a reduction in AC activity was reported in the hippocampus, temporal, frontal and occipital cortex, and cerebellum in AD conditions. Similarly, reductions in pathway effectors were also observed.^14,15^ These events are often paralleled by increased PDE activity in cerebrovessels, putamen, and temporal cortex (especially PDE5).^16,17^ However, AD-associated alterations in expression/activity are still debated, with contrasting data being reported and doubts about the cause–effect relationship between the events. Most importantly, these alterations appear to be isoform-specific and brain region-specific. As stated by Kelly in a recent review, “not all studies identify an upregulation of cAMP-PDE expression or activity in AD patient and model studies”, but preclinical and clinical evidence supporting the potential of PDE inhibitors is emerging.^11^ In similar ways, decreased cGMP signaling can be associated with aging. In particular, the involvement of cGMP in long-term synaptic activity in several brain regions should be taken into account.^18^

Concerning the involved downstream molecular mechanisms, variations in cAMP and cGMP signaling regulate the activation of cAMP response element binding protein (CREB), influencing the transcription of CRE-dependent genes and, thus, neurogenesis. By activating protein kinase A, cAMP also promotes CREB phosphorylation and consequently influences neuronal plasticity.^19,20^ Besides, it must be pointed out that NO itself is an effector of PDE activity of primary relevance.^21^ NO/cGMP signaling modulates synaptic transmission and plasticity in the hippocampus and cerebral cortex, which play critical roles in learning and memory.^22^ The NO/cGMP pathway induces CREB phosphorylation via protein kinase G, thus enhancing the effects described above connected to the cAMP-related signaling.^23−25^ Furthermore, NO activates the CREB pathway through the interaction with the NO receptor.^26^ Besides the effects on the CREB downstream, improved neurological outcomes related to the use of PDE inhibitors also result from a general NO-mediated enhancement of cerebral blood flow.^27^ Moreover, it has been observed that physiological concentrations of NO can also promote anti-apoptotic/pro-survival response against several neurotoxic insults through combined mechanisms.^22^ For a comprehensive overview of the role of NO in memory formation and in the regulation of cerebral blood flow to ensure adequate blood supply to the brain, the reader is invited to refer to the contribution by Maher and colleagues.^28^

Even if PDE inhibitors are generally prescribed for peripheral indications, their influence on intracellular signaling highlighted above makes them attractive tools for improving neuronal activity.^29−31^ Among the 11 PDE classes, isoforms 4, 7, and 8 specifically hydrolyze cAMP, isoforms 5, 6, and 9 are selective for cGMP, while the remaining enzymes can degrade both of these cyclic nucleotides.^1^ Details concerning the brain expression of such classes will be covered in the following paragraphs.

### 2.1 PDE5

**Figure 1:**
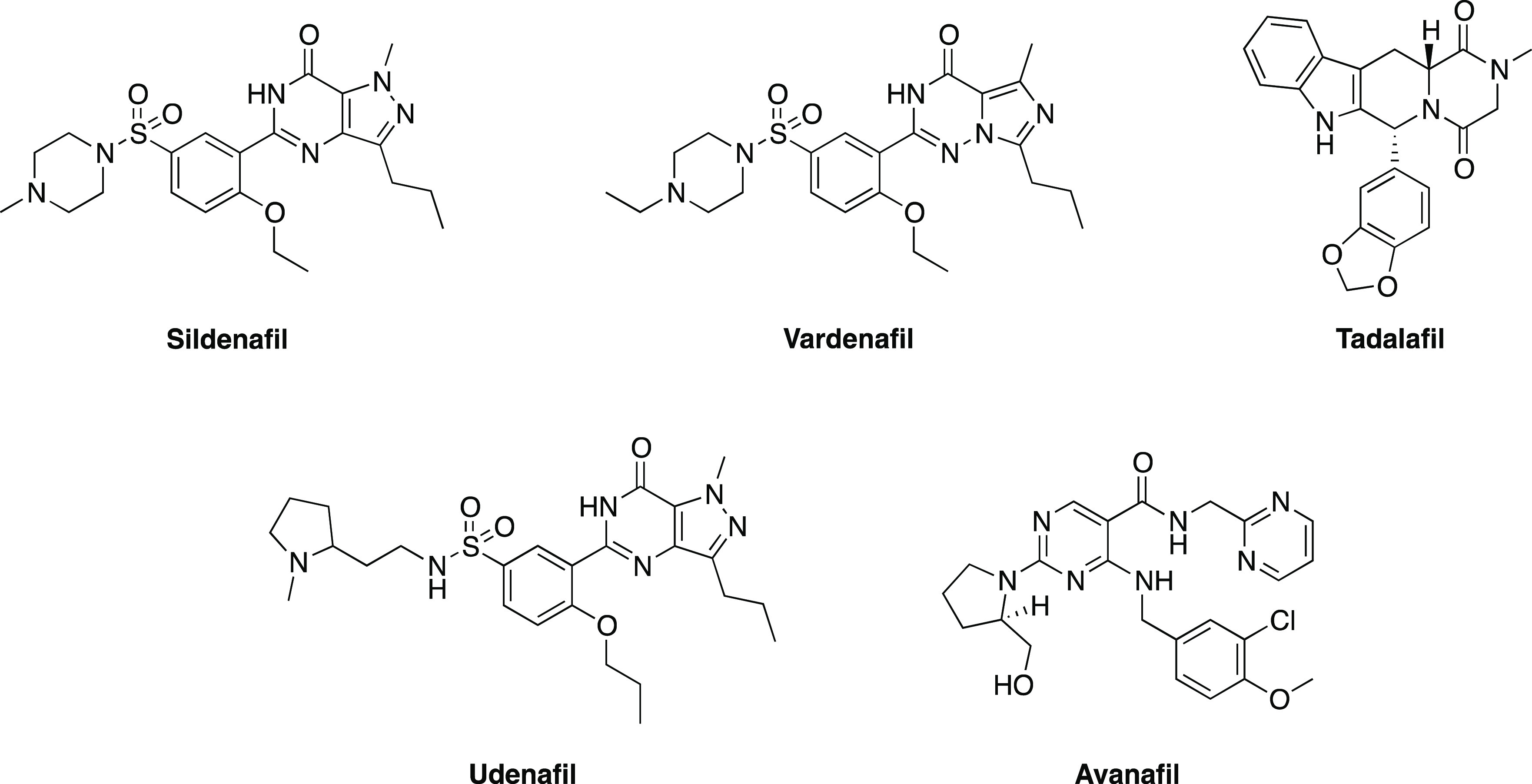
Chemical structures of commercially available PDE5 inhibitors.

PDE5 exerts its activity by selectively hydrolyzing cGMP to its corresponding linear derivative. It represents the pharmacological target of sildenafil and its analogues (Figure 1) in the treatment of erectile dysfunction (ED) and pulmonary hypertension (PH).^32^

The discovery of PDE5 localization in several human tissues paved the way for the investigation of novel possible therapeutic applications of selective PDE5 inhibitors. In this context, the fields of cancer, inflammation, and CNS-related diseases have been explored throughout the years.^10^ Gur et al. reviewed the evidence on the efficacy of PDE5 inhibitors in the latter research area, highlighting that such molecules have been studied to contrast epileptic disorders, stroke and cerebral ischemia, depression, and Huntington’s disease.^33^ More than two decades ago, the PDE5 inhibitor zaprinast was investigated for its beneficial effects on memory in the object recognition test. However, cross-reactivity with other PDE isoforms (1, 9, 10, and 11) limited its use. Thus, the attention of medicinal chemists is currently focused toward more selective inhibitors of natural and synthetic origin.

While it is now generally accepted that selective PDE5 inhibitors can promote beneficial effects on cognition and memory in both physiological and pathological conditions,^34^ the presence and localization of PDE5 in the human brain have been debated, especially considering the discrepancies highlighted with respect to rodent models. Nevertheless, PDE5 mRNA has been detected in human cortex, hippocampus, and striatum,^35^ and its distribution has been recently reassessed following the observations on the effectiveness of PDE5 inhibitors in contrasting AD in animal models.^34^ Interestingly, PDE5 expression is low or absent in the hippocampus of aged subjects and AD patients but is increased in the temporal cortex under similar pathologic conditions.^36^ In fact, mild AD patients show lower cGMP levels in cerebrospinal fluid (CSF).^37^ On the other hand, it must be stressed how, if sildenafil and its analogues are thought to exert an effect on central PDEs, the compounds should cross the blood–brain barrier (BBB), reaching the inhibiting concentration in the target tissue. It has been observed that both tadalafil and sildenafil may effectively accumulate in the brain at sufficient levels in animal models.^34,38^ This aspect will be discussed more in detail in the following section of this Review.

Sabayan et al. described PDE5 inhibitors as “disease-modifying agents” against AD and examined the molecular pathways through which the inhibition of such enzymes may play a role in contrasting the pathogenic process.^9^ In this context, the authors highlighted three main mechanisms that involve PDE5 and, thus, could be targeted by selective inhibitors. The first one is related to the “neurovascular theory”, based on the fact that microvascular injuries may reduce amyloid clearance through altered delivery of nutrients to neurons.^39^ Endothelial dysfunction has been highlighted in AD patients, even if the cause–effect relationship is not fully understood.^40^ Here relies the potential of PDE5 inhibitors, since they act by relaxing the arterial wall and improving endothelial functioning via the NO-cGMP pathway. By decreasing endothelin-1 and E-selectin levels, as has been observed in patients,^41^ they could act as disease-slowing agents. In the same context, there is evidence showing that a restored cerebral blood flow may improve glucose utilization and, in general, metabolic functions in neurons.^42^ The second mechanism is connected with the “cholinergic theory” of AD, and it is based on the mutual relationship between cGMP and acetylcholine in physiologic and pathologic conditions. In AD, acetylcholine levels are reduced in specific brain areas which are connected to memory and cognitive functions impairment. Consequently, two key points must be taken into consideration. First, cGMP is the second messenger of acetylcholine. Second, it has been observed that cGMP analogs can stimulate acetylcholine release.^43^ This sort of “double connection” suggests that stimulation of the NO-cGMP pathway induced by PDE5 inhibitors can result in an increased concentration of acetylcholine released by the *nucleus accumbens* and, thus, in memory enhancement due to the effects on cortical neurons.^43^ The third mechanism is related to impaired neurogenesis, a mechanism that underlies AD and other neurodegenerative diseases.^9^ Increased neurogenesis is considered a protective factor against AD, and it has been shown that in human adults this process occurs in olfactory bulbs and hippocampus. This is a crucial aspect, especially if the fact that neuronal progenitors in this area are located in close proximity to blood vessels is considered.^9,44,45^ Moreover, low levels of cGMP have been connected with decreased neuronal growth. As stated above, age-related reduction of cGMP may cause limited neurogenesis and, thus, impaired cognitive functions.^46^ It has been observed that sildenafil can revert this mechanism by stimulating progenitor cells’ proliferation in the hippocampus.^47^

From the biochemical point of view, multiple mechanisms underlie the three events promoted by PDE5 inhibitors described above. Sabayan and colleagues highlighted the role of enhanced CREB phosphorylation and the glutamate-NO-cGMP pathway: the NO-cGMP-protein kinase G pathway and overexpression/upregulation of the bcl-2 protein would be responsible for the anti-apoptotic effects in neurons.^9,48^

Concerning cognitive enhancement in humans, evidence on the role of PDE5 inhibitors is accumulating, even if with contrasting results in some of the cases. As an example, the cognitive status of ED patients without neurological or neuropsychiatric diseases treated with udenafil was tested: mini-mental and frontal assessment scores increased after administration (33 months), while the improvement in the Seoul learning test was not significant.^49^ The effects of sildenafil and tadalafil on cognitive functions have been studied more extensively in the past years, and the results are discussed in another section of this Review, while modest overall effects were observed for vardenafil.^50^

### 2.2 Other PDE Isoforms

**Figure 2:**
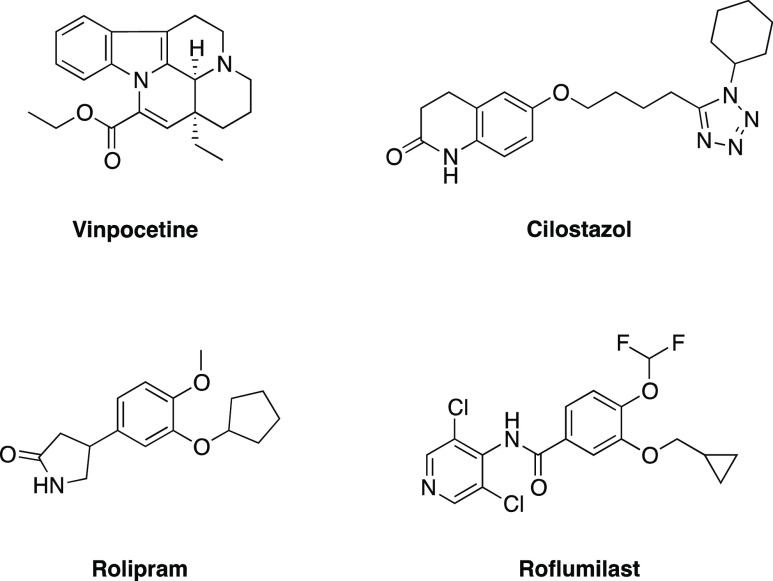
Chemical structures of the main inhibitors of other PDE isoforms.

PDE1, which is represented by different subtypes, is expressed in several brain areas such as the hippocampus, cerebral cortex, thalamus, and striatum 6 and 10.^1^ Vinpocetine (Figure 2) is a specific PDE1 inhibitor that was observed to improve memory and ameliorate streptozocin-induced cognitive dysfunction in rodent models.^51,52^ Interestingly, it has also been demonstrated that this compound can improve synaptic plasticity in a model of fetal alcohol spectrum disorders with impaired cortical development.^53^ Vinpocetine also gave positive results in other preclinical models and in cognitive tests in humans. However, despite encouraging results on healthy volunteers, further studies showed that vinpocetine failed to slow the decline of AD patients.^51,52^ Vinpocetine is nevertheless present in the Cognitex formulation, which was reported to have positive effects in an open label study.^54^ A possible development in the field of PDE1 is represented by a novel, selective inhibitor known as ITI-214. This compound improves memory acquisition, consolidation, and retrieval in rats, and Phase I clinical studies have been initiated (ClinicalTrials.gov Identifier: NCT01900522).^55,56^ Moreover, Dyck and colleagues recently presented a set of selective PDE1 inhibitors which showed promising results in enhancing memory in a rat model. Even more importantly, those authors reported the structure of the small molecule–protein complex.^57^

PDE2 is expressed in the cortex, amygdala, and hippocampus.^1^ It hydrolyzes both cGMP and cAMP, and it is the target of the inhibitor BAY 60-7550, which was observed to improve cognitive functions in rodents, and especially in memory-impaired rats and in a mouse models of AD.^58,59^ ND-7001 is another synthetic PDE2 inhibitor which was investigated for treating cognitive impairment but its development was then discontinued. Although PDE2 has attracted the interest of several pharmaceutical companies, no ongoing clinical trials have been reported.^3,60^

The dual-substrate enzyme PDE3 is expressed in the cerebellum, frontal cortex, hypothalamus, and hippocampus.^1^ Cilostazol (Figure 2) is a selective inhibitor of this isoform which enhances learning and ameliorates cognitive impairment in wild-type and AD model mice. As for PDE5 inhibitors, the effects appear to be the result of a combination of mechanisms of action, comprehending increased blood flow, and stimulation of the CREB pathway to promote synaptic plasticity.^61,62^ From the point of view of the clinical investigation as a potential target in AD treatment, PDE3 has been one of the earliest isoforms to be considered. In fact, several treatments and retrospective studies conducted on different populations of AD patients showed overall positive results, and the potential of PDE3 inhibitors in mild cognitive impairment (MCI) has also been demonstrated.^3^ In mild to moderate AD patients already on donepezil, cilostazol reduced cognitive decline.^63^ Another study investigated the effects of this inhibitor on cognition and regional cerebral blood flow. Cilostazol contrasted AD-related cognitive decline, but it has been observed that long-term treatment is required to obtain positive effects on blood flow.^64^ In a recent case-control study, cilostazol was tested as an add-on therapy for patients on AChE inhibitors, showing again positive results.^65^ Moreover, a retrospective study observed that daily administration of cilostazol decreases the risk of incident dementia.^66^ Nevertheless, side effects of PDE3 inhibitors must be taken into account: cilostazol may be dangerous for patients suffering from heart failure and severe hepatic or renal impairment.^3^

The cAMP-selective PDE4 isoform is widely expressed in the CNS,^67^ and in particular it is present in four subtypes and 20 variants.^68^ More specifically, PDE4D is more expressed in the CA1 hippocampal region and may be more closely involved in memory consolidation.^69^ Rolipram (Figure 2) and GEBR-7b target this isoform, while GSK356278 inhibits PDE4B.^1^ Rolipram has beneficial effects on hippocampal- and cortex-dependent memory, as demonstrated by several *in vivo* studies.^70^ However, its use in humans is limited by side effects such as severe headache and emesis. In this connection, studies on the more potent GEBR-7b and on allosteric modulators are emerging. For example, BPN14770 is a negative allosteric modulator of PDE4D well tolerated in Phase I studies.^34,71,72^ Similarly to rolipram, MEM 1414 entered Phase I but showed emetic effects.^73^ For other compounds which underwent or are undergoing clinical studies, such as MK-0952 and HT-0712, only limited and incomplete efficacy information is available.^3^ In addition to these examples, the FDA-approved roflumilast (Figure 2), an anti-inflammatory and anti-chronic obstructive pulmonary disease (COPD) agent, ameliorated impaired verbal learning performance in healthy adults. Interestingly, from the biochemical point of view, the activity of PDE4 inhibitors in contrasting AD appears to be amyloid-independent, since CREB activation represents the main mechanism.^3,74^ In a very recent contribution, Iraji et al. reported an overview of multi-target agents interacting with PDE4D as well as with other targets involved in neurodegeneration, such as cholinesterases, monoamine oxidase (MAO), and BACE1, which are relevant in AD onset and progression.^75^

PDE6 is only expressed in the pineal gland in the CNS, and thus it does not represent a target for contrasting neurodegeneration.^3^

The cAMP-selective PDE7 is expressed in the brain and has been studied as a target for ameliorating AD, in particular in combination with the activity of GSK3β. Its inhibition reduced cognitive impairment and pathological hallmarks in a mouse AD model.^76,77^ In a very recent contribution, Morales-Garcia et al. reported that genetic or small-molecule-mediated inhibition of PDE7 promotes neuroprotective and anti-inflammatory effects in several neurodegenerative disease models, with a special focus on Parkinson’s disease.^78^

PDE8 is another cAMP-specific phosphodiesterase and represents a potential target for contrasting AD that has been studied rather recently. While PDE8A expression is generally low in the human brain, it has been observed that PDE8B concentration increases in the hippocampus of AD patients. In particular, selective inhibitors such as PF-04957325 are being developed.^79,80^

PDE9 preferentially hydrolyzes cGMP, and its relevance as a target is testified by the fact that Pfizer and Boehringer entered Phase II trials with PF-04447943 and BI 409306 inhibitors, respectively.^81,82^ Safety, tolerability, dose proportionality, and relative bioavailability of tablet and oral solution formulations of the second compound were evaluated by Moschetti et al. in healthy human male subjects.^82^ Moreover, Li et al. investigated the potential of BAY 73-6691, a PDE9 inhibitor with good selectivity, on *in vitro* and *in vivo* models of AD.^83^ Nevertheless, it must be pointed out that compounds targeting PDE9 generally have a degree of inhibitory activity on other isoforms, which may result in undesired side effects.^1^

PDE10 is a dual-substrate enzyme that is mostly expressed in the striatum. Prickaerts et al. listed the clinical trials that several synthetic isoform-specific inhibitors underwent. However, given the limited results and PDE10 localization, pharmaceutical companies are currently re-evaluating the compounds for Huntington’s and Parkinson’s diseases.^3^

PDE11 is another non-specific enzyme, and it is the most recently identified isoform. According to *in vivo* studies, PDE11 seems to play a specific role in short-term and social memory. Even if some small molecules are being developed as inhibitors, no clinical investigation in this context has been reported.^84,85^

As a conclusion of this section of the review, focused on the inhibitors targeting PDE isoforms besides PDE5, relevant information on enzymes’ localization and studied compounds is summarized in Table 1. The reader is invited to refer to the recent review by Argyrousi et al. for more general information on PDE isoforms, genetic aspects, their localization in the human body, and their regulation.^86^

**Table 1:** Overview of PDEs’ Substrate Specificity, Localization in the CNS, Studied Inhibitors, and Relevant Literature References

isoform	substrate	localization in CNS	inhibitors	references
PDE1	cAMP/cGMP	hippocampus, cortex, thalamus, striatum	vinpocetine, investigational synthetic PDE1 inhibitors	Shekarian et al.,^52^ Deshmukh et al.,^51^ Medina et al.,^53^ Li et al.,^55^ Snyder et al.,^56^ Dyck et al.^57^
PDE2	cAMP/cGMP	hippocampus, cortex, amygdala	BAY 6007550, ND-7001	Reneerkens et al.,^58^ Sierksma et al.,^59^ Gomez et al.^60^
PDE3	cAMP/cGMP	cerebellum, frontal cortex, hypothalamus, hippocampus	cilostazol	Yanai et al.,^61^ Hiramatsu et al.^62^ Arai et al.,^63^ Sakurai et al.,^64^ Tai et al.,^65^ Tai et al.^66^
PDE4	cAMP	hippocampus, cortex	rolipram, GSK356278, BPN14770, MEM 1414, MK-0952, HT-0712, roflumilast	García-Barroso et al.,^1^ Prickaerts et al.,^3^ García-Osta et al.,^34^ Ricciarelli et al.,^71^ Burgin et al.,^72^ Gallant et al.,^73^ Van Duinen et al.^74^
PDE6	cGMP	pineal gland	–	Argyrousi et al.^86^
PDE7	cAMP	hippocampus, cortex, olfactory bulb, striatum, thalamus, hypothalamus, midbrain	investigational synthetic PDE7 inhibitors	Morales-Garcia et al.,^78^ Argyrousi et al.^86^
PDE8	cAMP	hippocampus, cortex, olfactory bulb, striatum, thalamus, hypothalamus, midbrain	PF-04957325	Pérez-Torres et al.,^79^ Vang et al.,^80^ Argyrousi et al.^86^
PDE9	cGMP	hippocampus, cortex, olfactory bulb, striatum, thalamus, hypothalamus, amygdala, midbrain, cerebellum	PF-04447943, BI 409306, BAY 73-6691	Schwam et al.,^81^ Moschetti et al.,^82^ Li et al.,^83^ Argyrousi et al.^86^
PDE10	cAMP/cGMP	striatum	–	Argyrousi et al.^86^
PDE11	cAMP/cGMP	low expression levels throughout the brain	–	Argyrousi et al.^86^

## 3 Focus on PDE5 Inhibitors against Neurodegeneration

In this section of the Review, a closer focus on some known PDE5 inhibitors of natural and synthetic origin will be provided.

### 3.1 Sildenafil

Developed as a novel tool against hypertension and angina pectoris, sildenafil was patented by Pfizer in 1996 and approved by the FDA in 1998 for the treatment of ED.^8^ During the following two decades, many novel investigational therapeutic applications were proposed for this compound, especially thanks to its overall safety.^10^ Sildenafil is reported to cross the BBB, and an indirect evidence of this feature is testified by some central side effects such as dizziness, headache, and vision changes.^33,87,88^ Nevertheless, it has to be pointed out that recent studies highlight a decreasing incidence of dementia in men in western countries, and better management of vascular conditions may be among the causes.^89^ In this context, sildenafil and PDE5 inhibitors may play a role and be considered as potential disease-modifying agents against AD that act mainly, but not only, by improving endothelial dysfunction.^9,90^

In more detail, many mechanisms through which sildenafil could contrast neurodegeneration were proposed through the years, and it was demonstrated that this compound can simultaneously act at different levels. One first event with which sildenafil can interfere is neurogenesis, or the birth of new neuronal cells. Neurogenesis occurs in adult forebrain regions of the subventricular zone and the dentate gyrus, and it is crucial for neuronal plasticity and, thus, for memory functions.^91^ It generally decreases with aging due to lowered cGMP production, as discussed in the previous section. Several reports (reviewed by Uthayathas et al.) show that sildenafil administration can promote neuronal cell proliferation by stimulating this pathway in rodent models,^8^ and these observations were recently confirmed by researchers using other PDE5 inhibitors.^92^ Moreover, sildenafil may enhance neurogenesis through Akt phosphorylation (as reported for tadalafil), which leads to an increased phosphorylation of the downstream target GSK3.^93^ This is a particularly relevant feature, given the outcome of the pathway on the secretase cathepsin B.^94^ Concerning other possibly involved molecular mechanisms, Orejana et al. observed that sildenafil decreases BACE1 expression in a SAMP8 mouse model and interferes with the Cdk5/p25 pathway.^94^ Memory enhancement is a crucial aspect, and in this context, the presynaptic and postsynaptic effects of sildenafil must be dissected. More specifically, presynaptic PDE5 inhibition increases cGMP concentration, promoting the release of glutamate and the activation of NMDA receptors. On the other hand, interference with postsynaptic PDE5 promotes a cascade leading to increased protein synthesis and synaptogenesis. Overall augmented activity of cGMP-coupled ion channels may help the early consolidation of memory.^95,96^ In this connection, Son et al. recently demonstrated that sildenafil exerts neuroprotective effects from mitochondrial toxicity induced by amyloid β (Aβ) peptide, and that ATP-sensitive potassium channels are involved. The authors concluded that sildenafil “suppresses the mitochondrial Ca^2+^ overload, disruption of mitochondrial functional integrity, and mitochondria-dependent apoptotic signaling in the HT-22 cells following Aβ-induced AD-like insult”.^97^

This mechanistic evidence is paralleled by experimental results on the efficacy of sildenafil in counteracting long-term memory deficit and focus attention in animal AD models and humans^96,98,99^ and recent data obtained studying other PDE5 inhibitors.^92^ Preclinical data support, to different degrees, the beneficial role of PDE5 inhibitors in promoting cognitive enhancements in rodents and primates.^34,100^ In mouse models, sildenafil administration was reported to ameliorate cognitive impairment and upregulate the brain-derived neurotrophic factor (BDNF), which contributes to the neuroprotective effects.^101,102^ The same compound also improved early consolidation processes and spatial memory (Morris water maze test, MWM).^103^ Moreover, the sildenafil-sustained NO-cGMP-protein kinase G pathway appears to be involved in enhancing learning and memory performance in rodents, and sildenafil administration also improved novel object recognition in rats with induced cognitive deficits.^42,98^

As previously stated, clinical studies on sildenafil and its effects on cognitive functions did not always show results in perfect agreement with *in vitro* or preclinical data.^96^ In general, sildenafil was not found to be effective on short-term memory, divided attention, and psychomotor tasks. On the other hand, improvements were observed in focused attention, simple reaction time tasks, and information processing in humans.^96,104,105^

Some sildenafil analogues were recently synthesized with the aim of simultaneously targeting PDE5 and histone deacetylase, another enzyme involved in the memory process, and tested *in vitro* and *in vivo* in a rodent model. This strategy aims at a synergistic effect, since PDE5 inhibition promotes CREB phosphorylation and the recruitment of CREB binding protein (CBP), which has histone acetylase activity.^106^ This path was very recently followed by Rabal et al., who presented a set of compounds structurally similar to sildenafil and its analogues that target PDE and histone deacetylase. The compounds, characterized by adequate ADMET and toxicity profiles, were tested by the authors on a mouse model of AD.^107,108^

Besides AD, sildenafil has also been tested for its activity on cognitive symptoms of schizophrenia, and no cognitive changes were observed when it was co-administered with anti-psychotics.^105^ In the context of other CNS-related diseases, the NO-cGMP pathway influences excitatory and inhibitory neurotransmission in epilepsy. Both pro- and anti-convulsant activities were reported for sildenafil, depending on the considered animal model and dose. Thus, the potential of PDE5 inhibition in this context remains rather unclear.^109^ More interestingly, psychotropic effects have been observed for sildenafil in humans with ED-related depression. Moreover, sildenafil showed promising results in behavior studies in rats in combination with atropine. It has also been highlighted that the anti-depressant-like effect of sildenafil may possibly proceed through the activation of oxytocin.^110,111^

### 3.2 Tadalafil

The repositioning of marketed drugs is recently becoming a commonly pursued strategy, since the safety of such agents has already been proved and their further development should be facilitated. As already stated, this may also be the case with PDE5 inhibitors, which are generally characterized by good pharmacokinetic properties and safety profiles. The evolution of clinical applications of tadalafil has been recently reviewed by N. S. Ahmed.^112^ Tadalafil can be safely administered orally on a daily basis, and this makes it a good candidate for repurposing as a memory enhancer.^113^ Tadalafil has been studied for its positive effects on cognitive processes by García-Barroso et al., who reported the effects of chronic treatment (5 weeks) on aged mice. According to the freezing behavior test, a measure of learned fear, tadalafil showed cognitive enhancements. It also promoted improvements in terms of escape latency. Moreover, those authors investigated the neuronal changes induced by this PDE5 inhibitor, showing that tadalafil administration is associated with an increase in the spine density on apical dendrites, which may be the mechanism underlying memory improvements. Anti-apoptotic pathways, such as those depending on Akt, could also be among the mechanisms of tadalafil in preventing neuronal cell death.^34^ To assess its pharmacokinetic properties, the compound was quantified in CSF by liquid chromatography–mass spectrometry (LC-MS): tadalafil levels in CSF after oral administration in non-human primates (2.4 mg/kg, given to *Macaca fascilurais*) were 1 order of magnitude higher than the IC_50_ for PDE5.^1,114^ Tadalafil was also previously reported to enhance spatial memory in the J20 AD mouse model more efficiently than sildenafil. The longer half-life, stability, and more efficient BBB permeability of the former may contribute to the observed effects.^34^

It is generally accepted that a chronic lack of blood flow to the deep brain area is common in, and may be related to the onset of, AD and other dementias.^115^ A clinical trial (ClinicalTrials.gov Identifier: NCT02450253) has been initiated to study the effects of tadalafil on patients with symptomatic small vessel disease in the brain but lacking the vascular cognitive impairment diagnosis, which would contribute to the understanding of the role of PDE5 inhibitors in contrasting the onset and progression of AD.^3,115^

Interestingly, it has been reported that tadalafil also possesses a certain degree of AChE inhibitory activity. Based on this observation and on the promising *in vivo* data discussed in this section, Mao et al. designed and synthesized a class of novel compounds inspired by the scaffold of tadalafil with dual AChE/PDE5 inhibitory activity and improved BBB permeability and solubility.^116^ This strategy was previously pursued by Zhou et al., who in a more preliminary study developed a class of multi-targeted, dual AChE/PDE5 inhibitors with good solubility and low cell toxicity.^117^ More recently, Ni et al. developed a set of water-soluble, drug-like, and BBB-permeable tadalafil analogues that showed good performance in ameliorating scopolamine-induced cognitive impairment. Such compounds were active on both AChE and PDE5 and induced enhanced CREB phosphorylation.^118^

### 3.3 Natural Compounds

**Figure 3:**
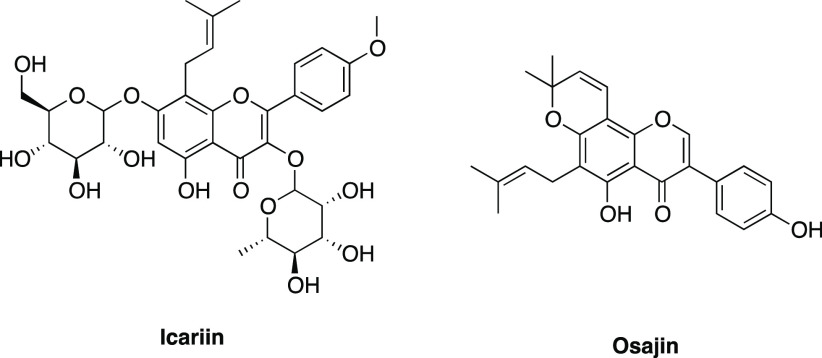
Chemical structures of some natural PDE5 inhibitors belonging to the class of flavonoids.

Several natural compounds have been described throughout the years as selective or non-selective inhibitors of PDE isoforms, and particular attention was dedicated to natural small molecules selectively inhibiting PDE5. Such compounds were often identified in plants traditionally used in folk medicine, mainly (but not only) as natural remedies for ED.^119,120^ Experimental evidence on the inhibitory effect of natural compounds from several different chemical classes ranges from *in silico* to *in vivo*.^121−125^ The potential of herbal extracts or isolated natural compounds with PDE inhibitory activity in contrasting AD progression was reviewed by Kumar et al. Those authors clearly listed several examples of natural plants with cAMP-specific, cGMP-specific, or dual inhibitory activity.^126^ The flavonoid icariin (Figure 3) is a known natural PDE5 inhibitor from *Epimedium brevicornum*. It represents an outstanding example from this class, since it has been shown to improve learning and memory functions in APP/PS1 transgenic mice. This effect likely occurs through the stimulation of the NO/cGMP signaling pathway, which results in decreased physiopathological changes in treated animals.^127^ Natural compounds often act through parallel mechanisms, and icariin is also an AChE inhibitor, which makes it an even more attractive candidate for the development of novel AD treatments.^128,129^

A multi-target mechanism was also demonstrated for natural and semi-synthetic isoflavones (Figure 3). This class of molecules, known for their PDE5 inhibitory activity, showed *in vitro* anti-AD effects on human recombinant BACE-1 inhibition assay and increased the activity of P-gp ATPase, with a possible role in the efflux of Aβ across the BBB.^122,130^

Several other examples of natural compounds with multiple mechanisms, involving PDEs inhibition at different levels, were previously reported. *Ginkgo biloba* extracts has been shown to produce anti-amyloid and anti-tau effects and to improve cognitive functions in dementia models. More specifically, the extract reduced tau phosphorylation in the C57BL6 mouse model.^126,131,132^ However, a clinical trial evaluating long-term use of standardized *Ginkgo biloba* extract did not highlight a decrease in the risk of progression to AD in comparison with placebo.^133^

Concluding, it must be stressed that PDE inhibition is not the only mechanism responsible for the neuroprotective activity of natural molecules, and several other biochemical pathways, even with direct effects on amyloid aggregation, were proven to be involved.^134−136^

## 4 The Point of View of the Medicinal Chemist: Multi-Target Approach and Drug-likeness

This Review originated from the need to provide an updated report on the state of the art of novel therapeutic options to contrast neurodegeneration based on PDE inhibition.^137^ While overviewing the past two decades of literature, we were prompted to rationalize and integrate available data using the tools of contemporary drug discovery. In particular, we must take into account that the interest in multi-target-directed ligands (MTDLs) is pushing the medicinal chemist to look beyond the usual “one molecule–one target–one disease” approach. Growing evidence shows that single drugs developed and extremely optimized to act on individual molecular targets are often inadequate or fail due to (unpredicted) side effects. One of the prominent current aims is based on the so-called “network pharmacology”, where the development of the drug candidate consists of combining, in a single small molecule, some multi-targeting properties.^138^ First, this applies to the study of non-selective PDE inhibitors (such as theophylline, resveratrol, and their derivatives), small molecules that act on different isoforms showing additive interactions and beneficial synergistic effects.^29,139,140^ In fact, as stated by Maurice et al., simultaneous inhibition of multiple PDEs leads to the activation of several signaling pathways, which may be desirable for treating complex diseases.^141^ In this context, the increasing understanding of the role of tissue and subcellular compartmentalization of cyclic nucleotides and individual PDE isoforms allows the development of more sophisticated strategies for interacting with such macromolecular targets.^141^

Then, on the side of isoform-selective inhibitors, the MTDL approach is being pursued in the study of novel remedies against dementia, as highlighted in the previous sections of this Review. This represents the real “core” of the rationale underlying the repurposing of PDE5 inhibitors such as tadalafil in this context. Recent contributions in the literature demonstrate the growing interest toward the identification of small molecules concurrently acting on PDE5 and on a secondary target involved in AD.^142^ This strategy would also aid the development of more targeted and personalized therapeutic approaches.^143^ In particular, the combination of the inhibitory effects on PDE5 and AChE is particularly attractive. In this context, a direct comparison between three of the most widely studied and promising PDE5 inhibitors discussed previously is provided below. Sildenafil, tadalafil, and the natural compound icariin have been compared basing on their inhibitory activity toward PDE5 and AChE and according to predicted pharmacokinetic properties, which are of primary relevance for CNS-acting drugs.

**Figure 4:**
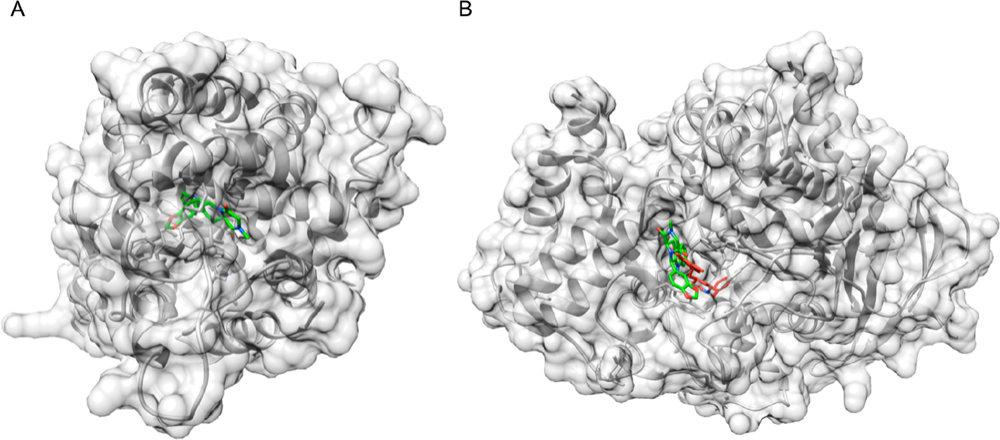
Crystal structure of the complex between tadalafil, depicted in green, and PDE5 (A). Calculated interaction pattern of tadalafil (green) with AChE; the originally co-crystalized ligand donepezil is represented in red (B).

Sildenafil is a potent PDE5 inhibitor (IC_50_ = 5.2 nM) but lacks effects on AChE.^117,144^ Tadalafil is again very effective on PDE5 (IC_50_ = 2.4 nM), while it inhibits AChE in the micromolar range (IC_50_ = 26.2 μM).^116,144^ On the other hand, icariin is a micromolar inhibitor of PDE5 (IC_50_ = 5.9 μM), but it is more effective in contrasting AChE activity (IC_50_ = 25.0 nM).^128,145^ Given these values, tadalafil appears as a promising dual-target candidate. Figure 4A represents the crystal structure of the tadalafil-PDE5 complex, and the ligand is highlighted in green. Docking studies were carried out to investigate the interaction pattern of the same small molecule with AChE, since a crystal structure of the complex is not available. Recent research works highlighted the reliability and significance of computational analysis modeling applied to the study of PDE5 inhibitors.^146^ The *in silico* experiments were carried out using AutoDock Vina, and the 3D structures were visualized with the UCSF Chimera molecular viewer.^147,148^ A brief description of the experimental protocol is reported in the following. Protein models were retrieved from the Protein Data Bank (www.rcsb.org, PDB ID: 1UDU for PDE5 in complex with tadalafil and PDB ID: 4EY7 for AChE in complex with donepezil). Target and ligands were prepared for the blind docking experiment performed using AutoDock Vina. Receptor grid generation was automatically set, thus encompassing the whole studied protein (blind docking). The number of docking poses was set to 10, and the other AutoDock Vina parameters were set to the default. Output data (energies and interaction patterns) were analyzed and scored using UCSF Chimera, which was also used to produce the artwork. Values reported in the following are expressed in kcal/mol and refer to the most favored pose. According to the predicted binding model, which was calculated by blind docking on the 3D structure of AChE, tadalafil interacts with the same binding site of donepezil (computed binding energy = −9.2 kcal/mol). To validate the protocol and as a positive control, donepezil was re-docked to the same protein (computed binding energy = −8.8 kcal/mol). A superimposition of the two compounds is represented in Figure 4B.

**Figure 5:**
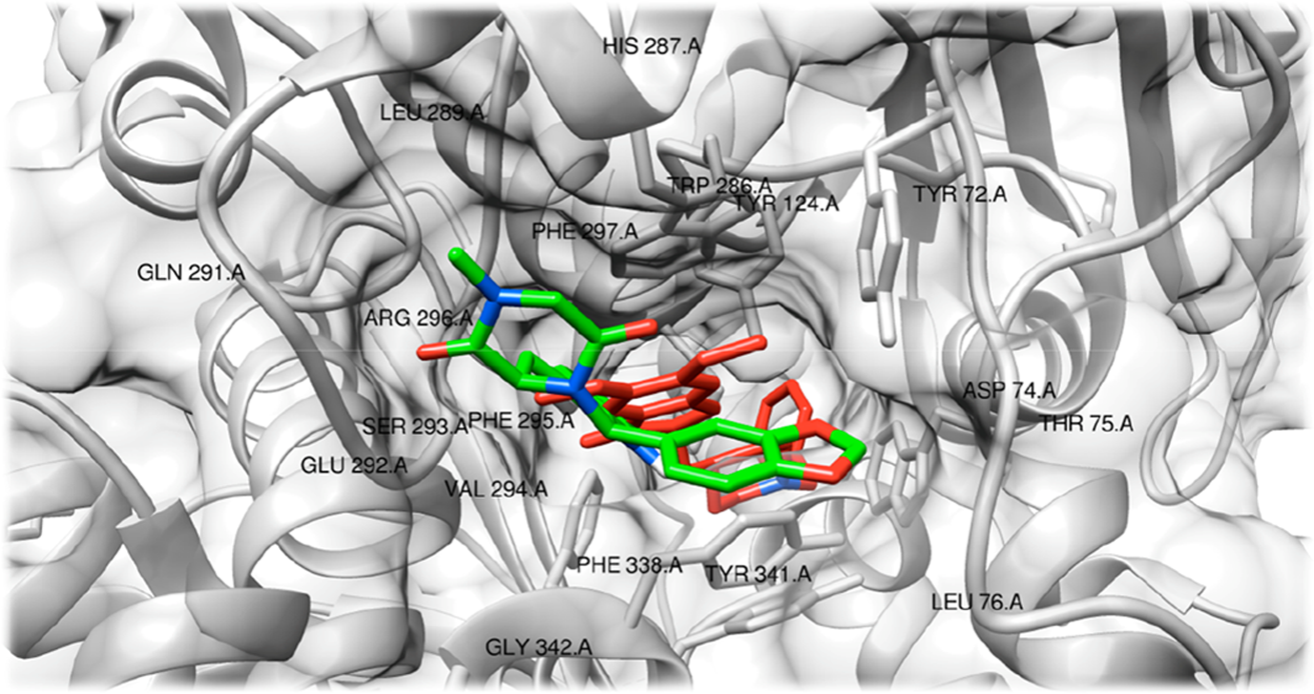
Detailed view of the AChE binding pocket. Calculated interaction pattern of tadalafil (green) is shown in comparison with the originally co-crystalized ligand donepezil, represented in red. Interacting residues (<5 Å) have been labeled.

A more detailed representation of the computed interaction pattern of tadalafil with AChE, in comparison with that of donepezil, is depicted in Figure 5. According to this docking study, the two compounds interact with the macromolecular target through the same residues. The amino acids that are located in the binding pocket (<5 Å from the ligand) have been labeled.

Besides the enzymatic activity data, the three compounds can be also compared from the point of view of their pharmacokinetic properties, which were calculated using Molinspiration Cheminformatics software (https://www.molinspiration.com). Topological polar surface area (TPSA), logP, volume, molecular weight, number of rotatable bonds, and numbers of hydrogen bond donors (nONH) and acceptors (nON) were computed and analyzed. Pajouhesh and Lenz efficiently determined the chemical and structural features of a successful CNS drug candidate:^149^ the reference values indicated by the authors are reported in the bottom line of Table 2, together with the values calculated for the three studied PDE5 inhibitors.

**Table 2:** Predicted Pharmacokinetic Properties for the Three Selected PDE5 Inhibitors^a^

	miLogP	TPSA (Å^2^)	no. of atoms	MW	nON	nOHNH	no. of violations	no. of rotatable bonds	volume (Å^3^)
sildenafil	2.51	113.43	33	474.59	10	1	0	7	419.47
tadalafil	2.36	74.88	29	389.41	7	1	0	1	334.03
icariin	1.67	238.21	48	676.67	15	8	3	1	582.92

values for CNS drugs		≤140 or ≤60 (ideal)	–	≤400	≤7	≤3	–	–	–

a Reference values for a successful CNS drug candidate are reported in the bottom line.

According to these drug-likeness criteria, tadalafil is the most promising CNS drug candidate of the set: its polar surface area is close to the ideal limit, and it is the only one not exceeding the molecular weight and number of hydrogen bond acceptor limits. Sildenafil has a higher polar surface area, which may limit BBB permeation, it has a molecular weight above 400, and its high number of heteroatoms pushes the value of hydrogen bond acceptors above the limit. Icariin is a very polar molecule and does not respect the limits proposed by Pajouhesh and Lenz. Another simple predictive rule states that BBB penetration is likely if the number of nitrogen and oxygen atoms is smaller than 5 ((N + O) ≤ 5).^149^ According to this formula, the computed values are 10 for sildenafil, 7 for tadalafil, and 15 for icariin. Thus, even if the desired value of 5 is exceeded by all the compounds, it can be stated that tadalafil is closer to this limit, again suggesting a better BBB permeability.

## 5 Conclusion

The selective inhibition of brain-expressed PDEs for contrasting neurodegeneration and dementia is a strategy that has been pursued for over two decades. PDE3, a dual-substrate enzyme, attracted a notable interest. Moreover, *in vivo* efficacy data on compounds targeting this isoform are already available, especially concerning the use of cilostazol. In addition to this, the attention that several pharmaceutical companies dedicated to the preclinical and clinical development of PDE4 and PDE9 inhibitors, belonging to different chemical classes, must be noted. The cGMP-selective PDE5 isoform is probably the most widely studied in this context, thanks to the availability of compounds that are already of clinical use and are known for their overall safety and good pharmacokinetic properties; *in vitro* and *in vivo* (preclinical and clinical) data currently focus on sildenafil, tadalafil, and some compounds of natural origin.

The involved molecular mechanisms underlying the activity of such inhibitors include CREB phosphorylation via protein kinase A or protein kinase B, depending on the involved PDE isoform, which would stimulate neuronal plasticity. Neuroprotective and anti-apoptotic effects in neurons are otherwise mediated by bcl-2 upregulation and the Akt-GSK3 pathway.

In conclusion, even if PDE inhibitors show very promising performances *in vitro* and in some *in vivo* disease model, contrasting results can be retrieved from clinical studies. Clinical trials with combined therapy appear to be more promising, and the multi-target approach may represent a favorable option. Taking advantage of “network pharmacology”, compounds with even modest activity on multiple, crucial targets could be optimized to tune their activity toward cognitive improvement. In this connection, early evidence is already emerging: the combination of PDE with AChE or histone deacetylase inhibitory activity is a promising strategy. The computational results here presented, together with the overviewed preliminary *in vitro* and *in vivo* evidence, suggest that tadalafil represents a promising starting point, in the context of PDE5 inhibitors, for the development of innovative tools to contrast neurodegeneration.

## Abbreviations

Aβ: amyloid β
AChE: acetylcholinesterase
AC: adenylyl cyclase
AD: Alzheimer’s disease
ADMET: absorption, distribution, metabolism, excretion, and toxicity
Akt: serine/threonine protein kinase B
BACE1: β-secretase 1
BBB: blood–brain barrier
BDNF: brain-derived neurotrophic factor
cAMP: 3′,5′-cyclic adenosine monophosphate
CBP: CREB binding protein
cGMP: 3′,5′-cyclic guanosine monophosphate
CNS: central nervous system
COPD: chronic obstructive pulmonary disease
CREB: cAMP response element binding protein
CSF: cerebrospinal fluid
ED: erectile dysfunction
FDA: U.S. Food and Drug Administration
GSK3: glycogen synthase kinase 3
LC-MS: liquid chromatography–mass spectrometry
MAO: monoamine oxidase
MCI: mild cognitive impairment
MTDL: multi-target-directed ligand
NMDA: *N*-methyl-d-aspartate
NO: nitric oxide
PDB: Protein Data Bank
PDE: phosphodiesterase
pGC: particulate guanylyl cyclase
P-gp: P-glycoprotein
PH: pulmonary hypertension
